# High-intensity intermittent cycling increases purine loss compared with workload-matched continuous moderate intensity cycling

**DOI:** 10.1007/s00421-014-2878-x

**Published:** 2014-04-19

**Authors:** Tracey Gerber, Melissa Louise Borg, Alan Hayes, Christos George Stathis

**Affiliations:** 1 College of Health and Biomedicine, Victoria University, PO Box 14428, Melbourne, VIC 8001 Australia; 2 Institute of Sport, Exercise and Active Living, Victoria University, Melbourne, Australia; 3 Present Address: Preventative Health, Baker IDI Heart and Diabetes Institute, Melbourne, Australia; 4 Present Address: Integrative Physiology, Karolinska Institutet, Stockholm, Sweden

**Keywords:** Hypoxanthine, Uric acid, Purine excretion, Energy expenditure, High-intensity intermittent exercise

## Abstract

**Purpose:**

Exercise at 50–60 % of peak oxygen consumption (*V*O_2 peak_) stimulates maximal fat oxidation rates. Despite a lower estimated work performed; high-intensity intermittent exercise (HIIE) training produces greater fat mass reductions when compared with workload-matched continuous (CON) steady state exercise. No metabolic basis has been documented nor mechanisms offered to explain this anomaly. This study investigated the physiological and metabolic responses of two different workload-matched exercise protocols.

**Methods:**

On separate occasions and at least 1 week apart, eight apparently healthy males cycled for 30 min at either 50 % *V*O_2 peak_ (CON) or performed repeated 20 s bouts of supramaximal exercise at 150 %*V*O_2 peak_ separated by 40 s rest (HIIE).

**Results:**

The average heart rate, oxygen consumption, plasma glycerol and free fatty acid concentrations were not different during exercise and recovery between the trials. Plasma lactate and hypoxanthine (Hx) concentrations were elevated and urinary excretion rates of Hx and uric acid were greater following HIIE as compared to CON (*P* < 0.05).

**Conclusion:**

Exercise-induced plasma Hx accumulation and urinary purine excretion are greater following HIIE and indirectly represents a net loss of adenosine triphosphate (ATP) from the muscle. The subsequent restorative processes required for intramuscular de novo replacement of ATP may contribute to a negative energy balance and in part, account for the potential accelerated fat loss observed with HIIE when compared with CON training programs.

## Introduction

Obesity rates across the world are reaching epidemic proportions and are associated with a wide variety of comorbidities, such as cardiovascular disease and type 2 diabetes (Willett et al. 1999). Lifestyle interventions (diet and exercise) are the most cost-effective strategies for reducing the risk of metabolic disease and remain the cornerstone for treatment options, however, most adults fail to meet the minimum physical activity guidelines, often citing a ‘lack of time’ as a key barrier to exercise participation (Trost et al. 2002; Reichart et al. 2007). Therefore, improving exercise participation and compliance rates by identifying alternate exercise strategies that provide health benefits and more effectively reduce adiposity are required (Metcalfe et al. 2012).

Traditionally, exercise for weight loss has focused on low to moderate intensity for long periods of time (>60 min) and are also reflected in recent guidelines from the American College of Sports Medicine that suggest 30 min of daily moderate exercise for health and weight loss (Garber et al. 2011). This ideal was centred on the metabolic basis that constant workload (CON) aerobic exercise, performed at around 50–60 % of maximal oxygen consumption (*V*O_2max_), induces maximal rates of fat oxidation relative to the rate of energy expenditure (Romijn et al. 1993). However, the long-term effectiveness on reducing body fat of CON exercise training programs has not been demonstrated (Shaw et al. 2006).

High-intensity intermittent exercise (HIIE) is characterised by repeated periods of intense exercise bouts interspersed with periods of passive recovery. It is a time efficient stimulus that induces physiological adaptations normally associated with continuous moderate intensity training (Gibala and McGee 2008) and HIIE training programs are more effective in their ability to improve fitness and health-related measures (Gibala et al. 2006; Rakobowchuk et al. 2008; Gibala and McGee 2008; Burgomaster et al. 2005, 2008; Richards et al. 2010; Gibala et al. 2012; Gunnarsson and Bangsbo 2012; Boyd et al. 2013) and more efficient at reducing adiposity (Tremblay et al. 1994; Trapp et al. 2008; Gremeaux et al. 2012; Macpherson et al. 2011) compared with CON exercise training.

It remains unclear as to how HIIE training decreases body fat when compared with the more traditional CON training programs. This is further complicated by the anomaly that the total HIIE training workload performed is lower when compared with CON training. A lower workload, as seen with HIIE, implies reduced energy expenditure impact on energy balance compared with CON training. However, the opposite trend in reduced adiposity was reported with HIIE compared with CON (Tremblay et al. 1994; Trapp et al. 2008; Gremeaux et al. 2012; Macpherson et al. 2011) suggesting higher energy expenditure and/or negative energy balance during the recovery period. As yet, no specific metabolic basis that accounts for this has been identified, however, the collective factors influencing postexercise oxygen consumption (EPOC) have been postulated (Boutcher 2011).

HIIE training has been shown to reduce resting ATP levels in the order of 20 % (Hellsten Westing et al. 1993; Stathis et al. 1994; Hellsten et al. 2004; Burgomaster et al. 2005). This metabolic consequence has been attributed to an elevated ATP turnover during the intermittent sprints and the acute degradation of ATP via a series of intramuscular reactions catalysed by AMP deaminase, 5′-nucleotidase and purine nucleotide phosphorylase to produce inosine monophosphate (IMP), inosine and hypoxanthine (Hx), respectively (Stathis et al. 1994, 1999, 2004); Hellsten et al. 1998. Hx effluxes the muscle (Hellsten et al. 1998) and is ultimately excreted in the urine as Hx or uric acid (UA) (Sorensen and Levinson 1975; Stathis et al. 1999, 2005). The elevated urinary excretion of Hx and its downstream metabolite, UA, are the end point of purine metabolism and are indirect markers of net ATP loss with exercise (Stathis et al. 1999, 2005). The replacement of the ATP lost from the muscle occurs via the purine de novo synthesis pathway during recovery and has an associated whole-body metabolic cost (Newsholme and Leech 1983) which is greater than intramuscular salvage. The purine base (Hx and UA) loss in the urine is influenced by exercise intensity and increases with successive sprints (Stathis et al. 1999) and may, in part, provide an explanation for the aforementioned anomaly between energy balance with HIIE and adiposity.

The present study compared the plasma profiles and urinary excretion patterns of several key substrates and metabolites during, and in recovery following, HIIE compared with a work-matched CON exercise bout. Differences in substrate and purine metabolism following single exercise protocols may elucidate possible metabolic mechanisms that will help explain the negative energy balance and fat loss in HIIE training. It was hypothesised that HIIE would result in elevated heart rate and oxygen consumption during exercise and recovery. Furthermore, plasma markers reflective of fat utilization will not be different between bouts; however, purine excretion rates postexercise (an indirect measure of ATP loss from the muscle) will be greater following HIIE as compared to CON.

## Methods

### Participants

Eight healthy untrained males aged between 18 and 35 years (22.4 ± 3.8 years; 76.6 ± 11.0 kg; 49.5 ± 5.7 mls·kg^−1 ^·min^−1^) volunteered to take part in this study, which was approved by the Victoria University Human Research Ethics Committee and performed in accordance with the ethical standards set out in the 1964 Declaration of Helsinki. After completing a medical questionnaire, each participant gave written informed consent and presented for preliminary testing.

### Preliminary testing

Peak oxygen consumption (*V*O_2peak_) was determined a minimum of 1 week before the beginning of the experimental trials. Participants completed a standard graded exercise protocol on a cycle ergometer (Lode, Groningen) of 3 × 3 min submaximal workloads at 50, 100, and 150 W followed by successive 1 min workload increments of 25 W until volitional exhaustion. Participants were encouraged to maintain a pedal frequency between 80 and 90 RPM and expired air was directed by a Hans Rudolph valve via a ventilometer into a mixing chamber and analysed for oxygen and carbon dioxide content (Applied Electrochemistry S-3A O_2_ and CD-3A CO_2_, respectively).

### Experimental protocols

In a randomised order, participants performed two exercise trials (HIIE and CON) separated by at least 1 week. The CON protocol involved steady-state continuous cycling for 30 min at a pre-calculated workload equivalent to 50 % *V*O_2 peak_ (127 ± 29.2 W). Based on power output, the HIIE protocol was matched for physical work completed over the 30 min and consisted of 1 min blocks of 20 s of exercise at three times the CON workload, i.e., 150 % *V*O_2peak_ (381 ± 87.7 W) separated by 40 s of passive recovery (i.e., work: rest was 1:2). All 30-min exercise periods were preceded by a rest period and followed by a 60-min recovery period where participants remained in a supine position. Participants were requested to refrain from strenuous exercise, caffeine, and alcohol 24 h prior to each experimental trial and maintained a 24 h food log to replicate food intake prior to the subsequent trial. All experimental trials were conducted in the morning to avoid influence of circadian variance, after an overnight fast of approximately 8–10 h.

### Heart rate analysis

Heart rate was monitored for the duration of the experimental trial and data was averaged over 5 min (equivalent to five intermittent bouts) of exercise for comparative purposes to account for oscillations during intermittent exercise.

### Respiratory gas exchange sampling and analysis

Expired air was sampled for 10 min in the supine position at rest, continuously during exercise, for 20 min immediately postexercise and the final 10 min of the 60-min recovery period. Data were sampled every 15 s, but was averaged over the 5 min.

### Blood sampling, treatment and analysis

A Teflon cannula was inserted into an antecubital vein, and was kept patent with isotonic saline (0.9 % NaCl, AstraZeneca, Port Melbourne, Australia). Blood was sampled at rest, during exercise at 1, 3, 5, 10, 15, and 30 min, and at 5, 10, 20, 30, and 60-min postexercise. Blood was immediately placed into either lithium heparin or ethylenediaminetetraacetic acid (EDTA; BD Vacutainer) tubes, mixed, centrifuged, and stored at −80 °C for later analysis. Plasma treated with lithium heparin was analysed for lactate and glucose (YSI 2300 STAT; Yellow Springs Instruments, Ohio, USA), and deproteinised before analysis for hypoxanthine and uric acid by reverse-phase high-performance liquid chromatography (HPLC) (Waters W600 Quaternary; Waters, US) and 996 Photodiode Array detector (monitored at 254 nm) using a Gemini C18, 5 μ (150 × 4 mm) analytical column. The mobile phase used for separation consisted of 10 mM hexanesulfuric acid (H_3_PO_4_, 0.5 %) and acetonitrile. The EDTA treated plasma was analysed spectrophotometrically for free fatty acids (FFA) (Wako, Japan) and glycerol (Sigma Aldrich, USA).

### Urine collection and analysis

Urine was collected overnight in the period prior to the trial and following 60-min recovery from exercise. Participants were instructed to consume water during recovery following exercise to ensure adequate urinary output. Total urinary volume was determined, with an aliquot stored at −80 °C before HPLC analysis of Hx and UA.

### Statistical analysis

The results are expressed as mean ± standard error of the mean (SEM). A two-way analysis of variance (time × exercise), with repeated measures was performed, separating the exercise and recovery periods (Graphpad Prism 6.0, California, USA). Where there was an interaction between the factors, a Bonferroni post hoc analysis was performed. Statistical significance was set at *P* < 0.05.

## Results

Oxygen consumption rose significantly with exercise to similar levels in both the HIIE and CON protocols, and returned to baseline after 10 min of recovery. Likewise, heart rate was significantly higher following exercise and was not different between the protocols, returning to baseline levels 20 min into the CON recovery period and remaining elevated for 30 min into the HIIE recovery period. The respiratory exchange ratio (RER) was significantly higher in the HIIE than the CON protocol during exercise (*P* = 0.005), and lower during the recovery period (*P* = 0.037).

**Fig. 1:**
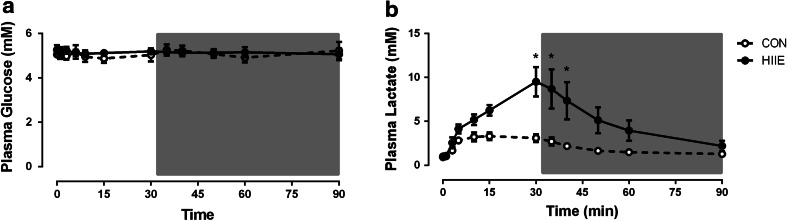
Plasma glucose and lactate during CON and HIIE exercise. Plasma glucose (**a**) and plasma lactate (**b**) measured during exercise and recovery from CON and HIIE. Data are mean ± SEM. *P < 0.05 CON vs. HIIE at a given time point, *n* = 8 per group. CON *white circles,* HIIE *black circles*. *Grey box* represents the recovery period

Plasma glucose concentrations were not different between trials and were maintained within a range between 4.95 and 5.26 mM for the duration of the two trials (Fig. 1a). Plasma lactate increased significantly from rest with exercise during the HIIE trial (*P* < 0.05) and was threefold higher in HIIE when compared with CON at the end of exercise (*P* = 0.0028) (Fig. 1b). Lactate in HIIE remained elevated above CON early in the recovery period (*P* = 0.039). Plasma glycerol was elevated during the exercise and recovery periods (*P* < 0.05 main effect), but was not different between trials. During the exercise, plasma FFA tended to be elevated in the HIIE trial (*P* = 0.06 main effect) and did not differ during the recovery period.

**Fig. 2:**
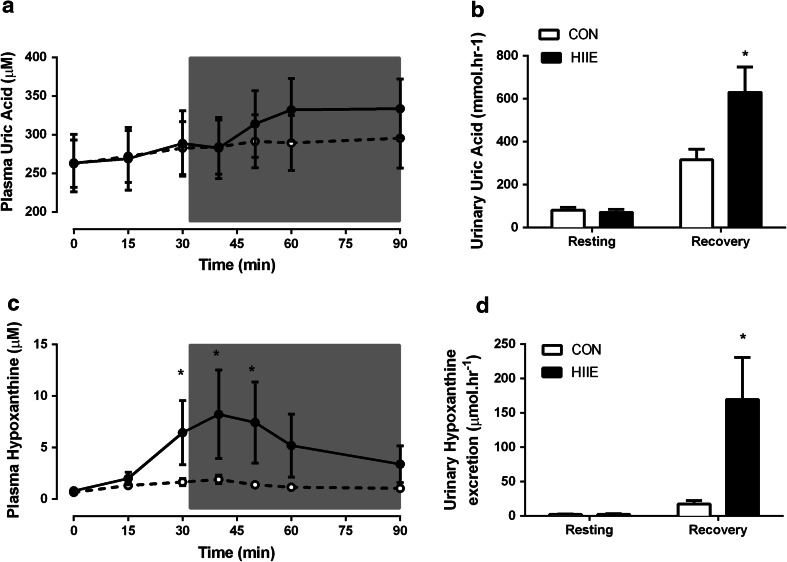
Purine metabolites during CON and HIIE exercise. Plasma uric acid (**a**) and plasma hypoxanthine (**c**) during exercise and recovery from CON and HIIE. Urinary uric acid excretion rate (**b**) and urinary hypoxanthine excretion rate (**d**) during resting (before exercise), and the recovery period (period including 30 min exercise and 60 min recovery). Data are mean ± SEM. *P < 0.05 Different from basal a given time point, *n* = 8 per group. CON *white circles*/*bars,* HIIE *black* *circles*/*bars*. *Grey box* represents the recovery period

Plasma UA remained constant during the CON exercise and recovery (Fig. 2a). However, plasma UA was significantly elevated above baseline levels at 20 (*P* = 0.03), 30 (*P* = 0.01), and 60 min (*P* = 0.01) after HIIE exercise. Urinary UA excretion was increased postexercise in both trials, however, CON resulted in a threefold increase in excretion from basal (*P* = 0.03), while HIIE resulted in an eightfold increase (*P* < 0.0001) in excretion rates (Fig. 2b). Plasma Hx increased over time in the HIIE (*P* = 0.025) and was significantly different from CON (*P* = 0.04) at completion of exercise (Fig. 2c). Plasma Hx remained elevated above baseline levels for 20 min into recovery (Fig. 2c; *P* = 0.004). HIIE resulted in a 70-fold increase in the urinary excretion of Hx (*P* = 0.003) whilst remaining constant during the CON exercise trial (Fig. 2d).

## Discussion

This study compared metabolic and physiological responses following workload-matched acute HIIE and CON exercise protocols. The major findings of this study were elevated plasma lactate and Hx concentrations following HIIE when compared with CON. This suggests a greater reliance on the glycolytic pathway and greater muscle metabolic stress, respectively, leading to significantly higher purine excretion after HIIE. The elevation in purine base excretion and the subsequent metabolic restorative cost of intramuscular ATP may, in part, provide a mechanism for a greater relative elevated energy imbalance and reduced adiposity in HIIE compared with CON training.

Higher plasma lactate levels with HIIE (Fig. 1b) are indicative of enhanced glycolytic contribution during HIIE exercise compared with CON. Furthermore, the combined profiles of plasma glycerol, plasma FFA and the RER during exercise and recovery suggest, albeit indirectly, that the extent of fat oxidation during exercise was not different between the two protocols. This is contrary to studies which show that with increasing exercise intensities there is increase in glycolysis but a reduction of fat oxidation rates (Romijn et al. 1993). Recent evidence suggests pyruvate from enhanced glycolytic activity results in increased acetyl CoA competing for the limited available carnitine in the mitochondria and consequently a reduced fat oxidation (Jeppesen and Kiens 2012). The glycolytic flux in HIIE in this study may not have been sufficient to influence the pyruvate accumulation and reduce the mitochondrial fat oxidation rates and may be a consequence of different physiological and metabolic responses due to the intermittent nature of the exercise.

The results from this current study are in line with an earlier paper (Essen et al. 1977), demonstrating no metabolic differences between an intermittent and continuous bout of exercise. This similarity in plasma profiles of both exercise bouts may be due to metabolic “yo-yo” effect the HIIE protocol creates on substrate partitioning with periods of elevated anaerobic glycolysis during an intense 20-s cycling bout, interspersed with oxidative recovery in the 40-s rest periods. Oxygen consumption increases with exercise and was not different between CON and HIIE trials. The RER oscillates in line with changes of the work and rest periods of the intermittent protocol and were greater early compared with later in the HIIE protocol (data not shown). However, the average values are not different between the trials (data not shown) and the plasma profiles of glycerol (data not shown) and FFA (data not shown) were not different and indirectly indicate a similar extent of fat oxidation in the two exercise bouts. Interestingly, these protocols were workload-matched and the greater metabolic perturbations produced by one metabolic system, i.e. glycolysis, during HIIE, were not reflected by a compensatory reduction in another, fat oxidation, albeit represented by indirect measures.

Furthermore, RER was significantly reduced in the early stages of recovery after HIIE. This decrease is more noteworthy as it occurs against a background of high plasma lactate and, by extension, an elevated plasma acid load, which would be likely to produce an elevated RER independent of substrate oxidation. This more rapid reduction of the RER immediately following HIIE as compared to CON exercise suggests a possible influence on the mechanisms involved in restoring bicarbonate buffering and/or an increased proportion of fat oxidation to support restorative processes during recovery. This is an interesting observation that lends itself to the potential for elevated fat oxidation rates in the recovery period following HIIE, supporting original observations linking intense exercise and reduced adiposity (Tremblay et al. 1994; Trapp et al. 2008; Gremeaux et al. 2012; Macpherson et al. 2011).

As discussed earlier, elevations in plasma Hx are the consequence of greater efflux from the muscle following exercise (Bangsbo et al. 1992; Hellsten et al. 1998; Stathis et al. 1994). Although plasma UA concentrations were not different between HIIE and CON trials, urinary purine (UA and Hx) excretion was at least two-fold higher in recovery post-HIIE when compared with CON (Fig. 2) and indirectly represents a greater loss of ATP from the muscle principally due to the enhanced metabolic stress of intense exercise (Stathis et al. 1999, 2006). Purine nucleotide metabolism is distinctly different between the skeletal muscle fibres types with a tendency for greater ATP degradation and subsequent purine loss in type II relative to type I fibres in response to intense exercise (Jansson et al. 1987; Karatzaferi et al. 2001). This may explain the significantly greater urinary excretion of Hx (Fig. 2b) and UA (Fig. 2d) after the shorter but more vigorous cycling periods compared with the lower intensity CON despite the same work output.

The energetics of replacing the purines from the muscle in recovery may influence energy expenditure relative to workload performed. The metabolic cost of replacing the intramuscular ATP stores, lost as Hx, via de novo synthesis pathway is slow and metabolically costly, estimated to require the equivalent energy of the hydrolysis of five ATP molecules compared with the intramuscular recovery of Hx to ATP via the purine salvage pathway (Newsholme and Leech 1983). It stands to reason that greater losses of Hx impose elevated metabolic energy costs for intramuscular ATP restoration following HIIE compared with CON, and provides a potential explanation for an enhanced energy imbalance and observed reductions in adiposity in earlier studies with supra maximal HIIE training compared with submaximal CON protocols (Tremblay et al. 1990, 1994; Trapp et al. 2008; Gremeaux et al. 2012; Macpherson et al. 2011).

The metabolic differences of HIIE indicated by increases in plasma lactate and purine urinary excretion rates postexercise were not reflected in elevated EPOC during the initial 60 min recovery period. Oxygen consumption was not different between the HIIE and CON protocols during the 30 min exercise or in the subsequent 60-min recovery period (data not shown). This result is contrary to the current concept that a greater metabolic perturbation of more intense exercise elevated oxygen consumption during exercise or recovery is more likely (Børsheim and Bahr 2003). However, it may be difficult to measure such metabolic alterations in terms of elevated oxygen consumption. The metabolic mechanisms of restoration generally occur over a long period of time during recovery and subtle differences may not be within the measurement capacity limits of elevated oxygen consumption. For example, the time course of recovery to resting ATP levels via de novo synthesis following a sprint training program is at least 48 h (Hellsten et al. 2004). A greater metabolism following HIIE is supported by Hazell et al. (2012) who report similar 24 h oxygen consumption following a single HIIE relative to CON protocol, despite the total oxygen consumption during the CON exercise being 150 % greater than the HIIE.

No difference in EPOC over the 60 min recovery period between the two protocols (Fig. 1a) indicates that in early recovery the factors influencing the fast phase of EPOC, i.e. re-saturation of myoglobin and haemoglobin, and the re-phosphorylation of ATP and PCr (Børsheim and Bahr 2003), was not different between the protocols. Intensity plays a role in elevation of the fast phase of EPOC (Børsheim and Bahr 2003) and as such HIIE in the current protocol may not have created an adequate metabolic disturbance. Another possibility is the opportunity for partial recovery of PCr during the intermittent rest periods (Bogdanis et al. 1996), potentially elevating O_2_ consumption during the HIIE rest periods, thus counteracting the anaerobic nature of the exercise itself. This may account for the similar O_2_ consumption observed between the two bouts over the 30 min exercise periods.

Unfortunately, EPOC was not measured beyond 60 min in this study to ascertain the influence of longer lasting components of EPOC such as elevated temperature, catecholamine levels and the replenishment of muscle glycogen (Børsheim and Bahr 2003). The restoration of purine bases also offers another mechanism for an elevated longer lasting component of EPOC. The oxygen consumption needs to be measured beyond 60 min into recovery to capture the potential elevation in metabolism due to both the resynthesis of intramuscular glycogen and ATP.

In summary, HIIE may stimulate similar and/or greater metabolic perturbations compared with workload-matched CON exercise. Plasma markers, although indirect, indicate no difference of fat metabolism between the trials. However, a more divergent response in plasma lactate and Hx levels indicate an elevated metabolic stress and altered muscle metabolism with HIIE. The elevated urinary excretion of purines following HIIE exercise is the end point of ATP degradation and hence the net excretion of purines over and above the basal rate can represent a greater loss of ATP from the active muscle. The extent of metabolic energy required for intramuscular restoration of this loss via de novo synthesis pathways is proportional to the magnitude of the loss. The loss of purine bases would be compounded by the repeated training sessions over time and consequently exacerbate the energy expenditure requirements during recovery of HIIE relative to CON training. This provides a mechanistic explanation to support the apparent paradoxical physiological observation that HIIE training, with lower total work outputs, reduces whole body adiposity to a greater extent compared with CON aerobic training. Furthermore, this evidence provides a metabolic basis for an enhanced fat loss utilising HIIE training for exercise prescription recommendations for public health guidelines and weight management practices for reducing body fat. This must be qualified as appropriate in young and healthy older populations, who can perform such exercise protocols without compromising cardiovascular safety limits or sustain injuries and results in better health outcomes in “time poor” modern lifestyles.

## Abbreviations

ATP: Adenosine triphosphate
CON: Continuous exercise
CRN: Collaborative research network
EDTA: Ethylenediaminetetraacetic acid
EPOC: Postexercise oxygen consumption
FFA: Free fatty acids
HIIE: High-intensity intermittent exercise
HPLC: High-performance liquid chromatography
Hx: Hypoxanthine
RER: Respiratory exchange ratio
SEM: Standard error of the mean
UA: Uric acid
*V*O_2 Peak_: Peak oxygen consumption
